# Evaluating and Correcting Inherent Bias of microRNA Expression in Illumina Sequencing Analysis

**DOI:** 10.3389/fmolb.2019.00017

**Published:** 2019-04-24

**Authors:** Anne Baroin-Tourancheau, Yan Jaszczyszyn, Xavier Benigni, Laurence Amar

**Affiliations:** ^1^Neuroendocrinologie Moléculaire de la Prise Alimentaire, Institut des Neurosciences Paris-Saclay (Neuro-PSI), CNRS UMR 9197, Université Paris-Sud, Université Paris-Saclay, Orsay, France; ^2^Institut de Biologie Intégrative de la Cellule (I2BC), CEA, CNRS UMR 9198, Université Paris-Sud, Université Paris-Saclay, Gif-sur-Yvette, France

**Keywords:** miRNA expression profile, high-throughput sequencing, Illumina technology, ligation bias, miRNA abundance, cerebellum

## Abstract

microRNA (miRNA) expression profiles based on the highly powerful Illumina sequencing technology rely on the construction of cDNA libraries in which adaptor ligation is known to deeply favor some miRNAs over others. This introduces erroneous measurements of the miRNA abundances and relative miRNA quantities in biological samples. Here, by using the commercial miRXplore Universal Reference that contains an equimolar mixture of 963 animal miRNAs and TruSeq or bulged adaptors, we describe a method for correcting ligation biases in expression profiles obtained with standard protocols of cDNA library construction and provide data for quantifying the true miRNA abundances in biological samples. Ligation biases were evaluated at three ratios of miRNA to 3′-adaptor and four numbers of polymerase chain reaction amplification cycles by calculating efficiency captures/correcting factors for each miRNA. We show that ligation biases lead to over- or under-expression covering a 10^5^ amplitude range. We also show that, at each miRNA:3′-adaptor ratio, coefficients of variation (CVs) of efficiency captures calculated over the four number of amplification cycles using sliding windows of 10 values ranged from 0.1 for the miRNAs of high expression to 0.6 for the miRNAs of low expression. Efficiency captures of miRNAs of high and low expression in profiles are therefore differently impacted by the number of amplification cycles. Importantly, we observed that at a given number of amplification cycles, CVs of efficiency captures calculated over the three miRNA:3′-adaptor ratios displayed a steady value of 0.3 +/− 0.05 STD for miRNAs of high and low expression. This allows, at a given number of amplification cycles, accurate comparison of miRNA expression between biological samples over a substantial expression range. Finally we provide tables of correcting factors that allow to measure the abundances of 963 miRNAs in biological samples from TruSeq-based expression profiles and, an example of their use by characterizing miRNAs of the let-7, miR-26, miR-29, and miR-30 families as the more abundant miRNAs of the rat adult cerebellum.

## Introduction

RNA-Seq has become very popular in analyses of RNA expression. In < 10 years, the RNA-Seq Illumina technology became a technology of choice because of its affordability and powerful resolution. New sequencing technologies are now available (Garalde et al., 2018). They however concern RNA-seq rather than microRNA-seq (miRNA-seq). Indeed the very short size of microRNAs (miRNAs), around twenty nucleotides, does not stand the current level of sequencing error of those new technologies and impedes the discrimination of miRNAs of related sequences but distinct functions. Many studies tend to consider a high miRNA expression in sequencing data as an indicator of a high miRNA amount in biological samples as if miRNA expression profiles faithfully represent starting miRNA populations in terms of diversity (distribution of miRNAs) and proportions (relative abundances of miRNAs). If the former is mainly (but not entirely, see below) a matter of sequencing depth, the latter is not true. This major pitfall that was pointed out in pioneer works (Linsen et al., 2009; Hafner et al., 2011; Sorefan et al., 2012; Zhuang et al., 2012) has not since then received proper attention in the research community. As miRNA relative expressions in profiles are very different from miRNA relative amounts in starting materials, confusion between both impedes the valid selection of miRNAs of interest and design of experiments in physiological/physiopathological research.

miRNA relative expressions in miRNA-seq-based profiles display discrepancies with miRNA relative amounts in samples as a consequence of inherent biases introduced during cDNA library constructions whatever the adaptors/commercial kit used (NEB Next, NEXTFlex, SMARTer, CATS, and TruSeq) (Baran-Gale et al., 2015; Yeri et al., 2018). When using the RNA-seq Illumina technology, the construction of cDNA libraries involves the ligation of an adaptor at each miRNA end, reverse-transcription of the resulting chimeric molecule into a single-stranded cDNA and its amplification into double-stranded cDNAs with a polymerase chain reaction (PCR). A recent and detailed analysis of the molecular basis of expression biases in miRNA expression profiles demonstrated that the ligations of the 3′-adaptor and 5′-adaptor to miRNAs are responsible for this (Fuchs et al., 2015). Ligation reactions have to be performed at 15°C−30°C, a temperature range at which different miRNAs display large conformational differences. Depending on the conformation, the ligation of the miRNA and adaptors is more or less favored. As a consequence, some miRNAs are over- or under-represented in cDNA libraries and the relative proportions of cDNAs do not reflect the relative proportions of miRNAs.

Our motivation here was to evaluate the level of biased expression of miRNAs in profiles established using the classical TruSeq protocol of the Illumina technology and do this at different miRNA amounts and different numbers of amplification cycles of the PCR. For 963 animal miRNAs, we established factors that significantly corrected bias/distortions of expression. The use of these correcting factors enabled us to assess miRNA abundances in the rat adult cerebellum.

## Materials and Methods

### RNA Sample

The equimolar mixture of 1006 RNA oligonucleotides corresponding to 43 RNA calibrators and 963 miRNAs (miRXplore Universal Reference, Myltenyl Biotec) was resuspended in RNase-free H2O (Supplemental Table 1).

### Construction of cDNA Libraries

All the DNA and RNA oligonucleotides used in the preparation of cDNA libraries were purchased from Sigma (France) (Supplemental Table 2). The 3′-adaptors were bought phosphorylated at the 5′ end and blocked at the 3′ end by a C7-Amine residue. They were adenylated as described (Vigneault et al., 2008). The construction protocol encompassed three steps: 1/ Two, 20 or 200 fmoles of RNAs were added to 125 fmoles of the adenylated RA3 3′-adaptor of the current TruSeq Illumina-based protocols or BC8 3′-adaptor of early Illumina-based protocols (Baroin-Tourancheau et al., 2016). This defined ratios of RNA:3′-adaptor close to 1:0.6, 1:6 or 1:60. The mixtures were incubated 3 min at 70°C, then added with 2.4 μl of PEG 8000 (Biolabs), 10X truncated T4 RNA ligase 2 buffer and 0.4 μl (20 U/μl) of T4 RNA ligase 2 truncated K227Q (Biolabs) up to a final volume of 10 μl and incubated for 75 min at 25°C. The excess of the 3′-adaptor was subsequently sequestered by the addition of 25 pmoles of the RT-primer and successive incubations of the mixture 3 min at 70°C, 15 min at 37°C and 15 min at 25°C. The mixture was then added with 1 pmole of the 5′-adaptor, 10 nmoles of ATP and 1.0 μl (20 U/μl) of T4 RNA ligase 1 (Biolabs) and incubated for 60 min at 25°C; 2/The chimeric molecules were converted to single stranded cDNAs using 130 U of Superscript III reverse transcriptase (Life technology) at 50°C for 90 min in a final volume of 30 μl; 3/Following reverse transcription, the RT reaction was added a PCR mix that contained 30 μl of 2X Master Mix Phusion enzyme (Biolabs), 60 pmoles of primer PCR-F and 120 pmoles of primer PCR-R. The resulting 60 μl reaction volume was distributed into 4 tubes in order to enhance temperature exchange efficiency, denaturated for 1 mn at 98°C and submitted to 16, 20, 24, or 28 amplification cycles (20 s at 98°C, 30 s at 55°C, 25 s at 72°C). The PCR products were added 6 ul of sodium acetate 3M, EtOH precipitated, rinsed and resuspended before size-fractionation on a 6% TBE polyacrylamide gel to purify RNA-derived cDNAs from adaptor dimers. cDNAs were eluted overnight in 0.4M NaCl, EtOH precipitated and resuspended in 10 ul of RNase-free H2O.

### Sequencing Data Analysis

Barcoded cDNA libraries were pooled together and sequenced on a NextSeq sequence lane at the sequencing platform of I2BC (https://www.i2bc.paris-saclay.fr). The reads from the cDNA libraries constructed using the RA3 3′-adaptor were obtained demultiplexed and trimmed from the 3′-adaptor. The reads from the cDNA libraries constructed using the BC8 3′-adaptor were demultiplexed, filtered and trimmed from the adaptor using our script written for this purpose. More than 90% of the reads were 19–25 nucleotides-long thereby reflecting the size of the sequences of the RNA oligonucleotides of the miRXplore Universal Reference. Reads were mapped onto the repertoire of sequences of the miRXplore Universal Reference using the BWA alignment program allowing 0 mismatch (Li and Durbin, 2009). The raw data files are available at the SRA database (NCBI) under the accession numbers (SRX5059960-5059974, SRX363287-90, SRX363303-18).

## Results

### Characterizing Distorsions of miRNA Expression in Illumina-Based Profiles

In order to identify miRNA over- or under capture efficiencies, we have built 12 cDNA libraries from the miRXplore Universal Reference consisting of equimolar amounts of 1006 synthetic RNAs of around 20 nucleotides. 43 and 963 of those RNAs are calibrators and animal miRNAs, respectively. Out of the latters, 102 and 861 mimic viral and cellular miRNAs, respectively.

We first used the current TruSeq/Illumina protocol (see Materials and Methods). Previous work had shown a high similarity between expression profiles established from technical duplicates that had been built with one given RNA amount and one given number of amplification cycles, Pearson correlation coefficients R^2^ being higher than 0.97 (Fuchs et al., 2015). For this reason, we constructed 12 singular cDNA libraries differing one from the other in the ratio of RNA:3′-adaptor (1:0.6, 1:6, or 1:60) and/or in the number of amplification cycles (16, 20, 24, or 28) (Supplemental Figure 1). As 963 of the 1,006 RNAs of the miRXplore Universal Reference are miRNAs, we referred to RNA:3′-adaptor ratios as miRNA:3′-adaptor ratios. Ratios of miRNA:3′-adaptor and numbers of amplification cycles ranged within those commonly used when working with biological samples (see below).

All cDNA libraries were sequenced at a depth of around two millions of reads except one for which less than 10,000 reads were recovered (Supplemental Figure 2). The reads were mapped onto the repertoire of the miRXplore Universal Reference to construct expression profiles (see Materials and Methods). More than 90% of the reads mapped to the full-length sequences while 10% of the reads mapped to sequences shorter by one or a few nucleotides. Those reads identified molecules that probably result from interrupted oligonucleotide chemical syntheses (see Discussion). For each RNA, reads of full-length and shorter sequences were summed. Two to Eight Percent of the 1,006 RNAs, mainly the RNA calibrators, were almost absent from the expression profiles. RNAs of the miRXplore Universal Reference are referred below as miRNAs.

**Figure 1:**
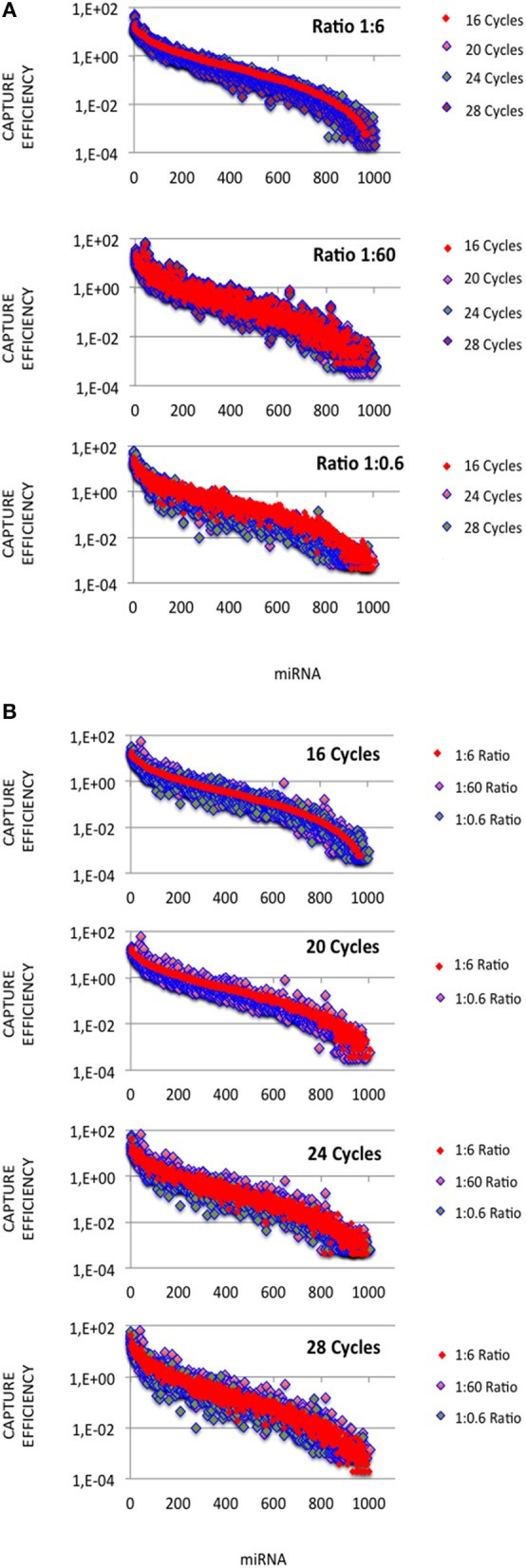
miRNA capture efficiencies extend over a 10^5^ amplitude range. cDNA libraries were built from equimolar miRNA amounts with ratios of miRNA:3′ adaptor of 1:0.6, 1:6 or 1:60, and 16, 20, 24, or 28 amplification cycles. For each miRNA:3′ adaptor ratio and each amplification cycle number, we quantified the efficiency of capture (EC) of each miRNA by dividing the miRNA expression frequency (i.e., miRNA reads/sum of miRNA reads) by the miRNA abundance in the sample (i.e., 1/1006). ECs are plotted for different miRNA:3′ adaptor ratios **(A)** or different amplification cycle numbers **(B)**. miRNAs were identically ordered on the X-axes of the plots of **(A,B)**. This order was obtained by sorting ECs calculated from the expression profile established when using the miRNA:3′ adaptor ratio of 1:6 and 16 amplification cycles (see Supplemental Table S3). Y-axes are drawn using a log_10_ scale. Note that the values of ECs largely overlap in each plot.

**Figure 2:**
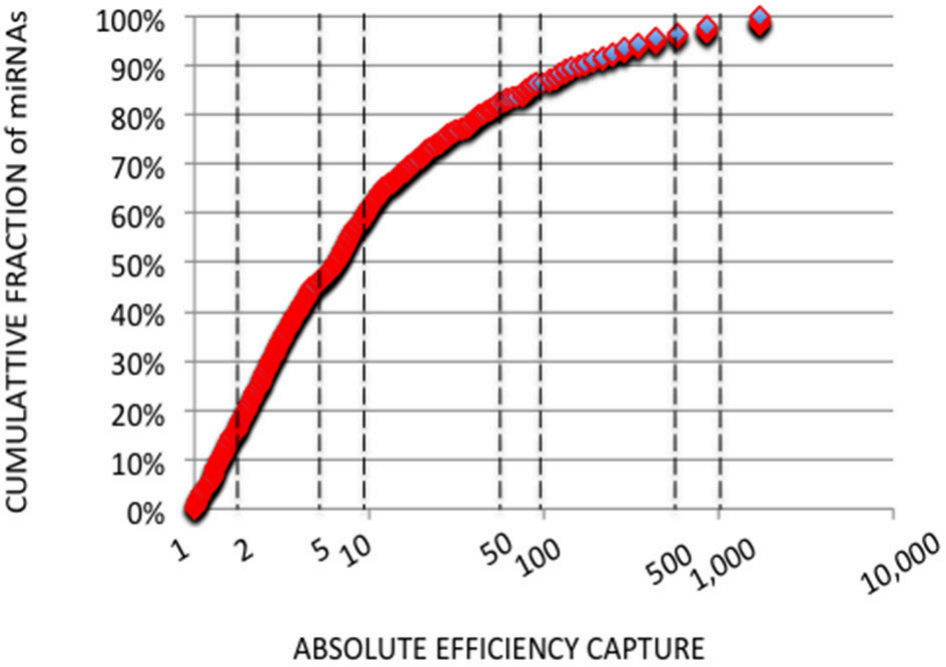
Cumulative Analysis of miRNA Capture Efficiencies. Cumulative fractions of miRNA (Y-axis) were plotted against *absolute* ECs (X-axis) calculated from the expression profile with a miRNA:3′ adaptor ratio of 1:6 and 16 amplification cycles. *Absolute* ECs were defined as maximal values between ECs and 1/ECs so that over- and under-expressions are collapsed (ECs of 50 and 0.02 for example). The X-axis is drawn using a log_10_ scale. About 60 and 85% of the miRNAs displayed *absolute* ECs lower than 10 or 100, respectively. About 40 and 15% of the miRNAs displayed *absolute* ECs higher than 10 or 100, respectively, and would artifactually appear as over- or under-abundant.

In each expression profile, for every miRNA, we established an efficiency of capture (EC) by dividing the miRNA expression frequency (i.e., full-length and shorter reads of each miRNA/total number of miRNA reads) by the miRNA abundance in the miRXplore Universal Reference (i.e., 1/1,006) (Supplemental Table 3). In each expression profile, ECs displayed a sigmoid curve that extended over a 10^5^ amplitude range across the miRNAs, from 2E-4 to 60 (Figures 1A,B). A large number of the *absolute* ECs that were defined as the maximal values between EC and 1/EC for each miRNA were higher than 10 (i.e., ECs higher than 10 or lower than 0.1) or 100 (i.e., ECs higher than 100 or lower than 0.01). For instance, at the miRNA:3′-adaptor ratio of 1:6 and for 16 amplification cycles, 60 and 85% of the miRNAs displayed an *absolute* EC lower than 10 and 100, respectively, so that 40 and 15% of the miRNAs displayed an *absolute* EC higher than 10 or 100, respectively (Figure 2).

The miRNA:3′-adaptor ratio is a parameter difficult to control between biological samples. In some cases, additional rounds of amplification are required to get a cDNA library of a sufficient size. For each miRNA, we therefore investigated whether ECs were affected by the miRNA:3′-adaptor ratio and/or number of amplification cycles. To do this, we characterized Pearson correlation coefficients R^2^ of ECs between different miRNA:3′-adaptor ratios or different numbers of amplification cycles. In addition, we calculated the coefficients of variation (ratio of standard deviation to mean, CV) for ECs characterizing one miRNA:3′-adaptor and different numbers of amplification cycles or, one number of amplification cycles and different miRNA: 3′-adaptor ratios. For those calculations, we considered the miRNAs defined by the top 800 ECs that were higher than 0.01 when using a miRNA:3′-adaptor ratio of 1:6 and 16 amplification cycles (see Figures 1A,B). ECs lower than 0.01 corresponded to miRNAs identified by < 10 reads per million (RPM) in profiles, a count too low to ensure accurate quantification of miRNA expression.

**Figure 3:**
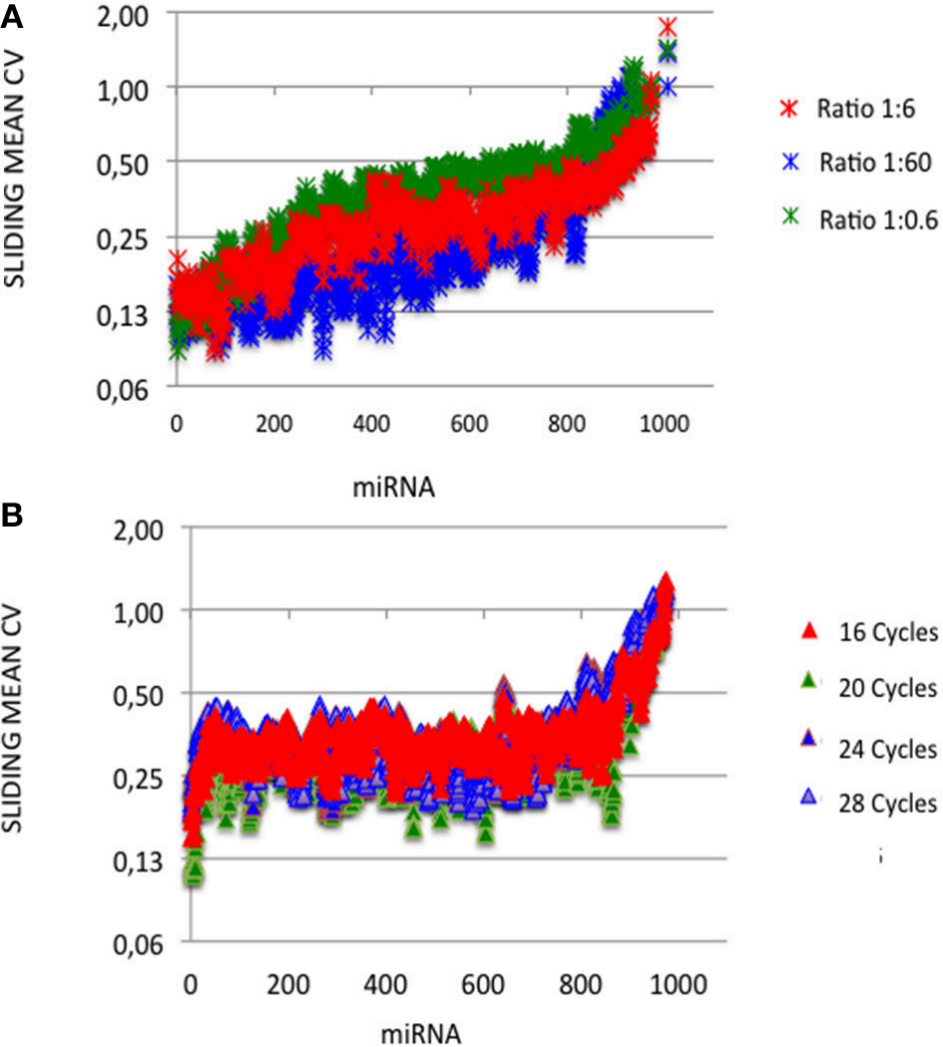
Capture efficiency robustness. We analyzed the robustness of ECs by plotting mean coefficients of variation (CVs) of ECs calculated built over sliding windows of 10 values using different miRNA:3′ adaptor ratios **(A)** or different amplification cycle numbers **(B)**. miRNAs were ordered as in Figures 1, 2. Y-axes are drawn using a log_2_ scale. Note that the values of ECs largely overlap in each plot.

At each miRNA:3′-adaptor ratio, the number of amplification cycles had a low impact: the Pearson correlation coefficients R^2^ between ECs calculated for 16 cycles on one hand and ECs calculated for 20, 24, or 28 cycles on the other hand were higher than 0.82 (Table 1) (see below). At each miRNA:3′-adaptor ratio, CVs of ECs calculated over the four number of amplification cycles ranged from 0.1 to 0.6, from miRNAs of high expression to miRNAs of low expression (Figure 3A). ECs of miRNAs of high and low expression in profiles therefore appeared differently impacted by the number of amplification cycles with the former being about 6-fold less impacted than the latter.

**Table 1:** Capture efficiency robustness against differences in amplification cycle numbers.

	**16 cycles**		
	**vs**.		
**miRNA: 3^**′**^ adaptor ratio**	**20 cycles**	**24 cycles**	**28 cycles**
1:0.6	ND	0.93	0.93
1:6	1.00	0.82	0.82
1:60	1.00	0.95	0.92

*Pearson coefficients of determination R^2^ were calculated at miRNA:3′-adaptor ratios of 1:0.6, 1:6, and 1:60 between ECs established with 16 amplification cycles on one hand and ECs established with 20, 24, or 28 amplification cycles on the other hand. For those calculations, we considered the top 800 ECs that were higher than 0.01. miRNAs with ECs lower than 0.01 were identified by < 10 reads per million (RPM), a current elimination threshold for accurate quantification of miRNA expression*.

At a given number of amplification cycles, the Pearson correlation coefficients R^2^ between ECs calculated for the 1:6 ratio on one hand and ECs calculated for the 1:0.6 or 1:60 ratio on the other hand ranged from 0.46 to 0.87 (Table 2). ECs appeared thus more sensitive to the miRNA:3′-adaptor ratio than to the number of amplification cycles. The lowest Pearson correlation coefficient R^2^ of 0.46 characterized the comparison of the ECs of the 1:6 and 1:60 miRNA:3′adaptor ratios for 24 amplification cycles. To estimate the biological meaning of this R^2^ value, the EC of each miRNA in the 1:60 ratio was plotted against its corresponding EC in the 1:6 ratio (Supplemental Figure 3). Even in that case, the ratio of the ECs of the 1:6 and 1:60 miRNA:3′adaptor ratios displayed less than a 4-fold difference for all miRNAs but 19 of them. The level of this difference was on an altogether different level than the one characterizing the different miRNAs of the miRXplore Universal Reference (see above). Finally we observed that the CVs of ECs calculated over the three miRNA:3′-adaptor ratios displayed a steady value of 0.3 ± 0.05 STD indicating that ECs of miRNAs of high and low expression were not differently impacted by the miRNA:3′-adaptor ratio (Figure 3B).

**Table 2:** Capture efficiency robustness against differences in miRNA:3′-adaptor ratios.

	**miRNA: 3**^****′****^ **adaptor ratio of 1:6**	
	**vs**.	
**Cycles**	**Ratio 1:0.6**	**Ratio 1:60**
16	0.87	0.60
20	ND	0.56
24	0.90	0.46
28	0.89	0.49

*Pearson coefficients of determination R^2^ were calculated at 16, 20, 24 or 28 amplification cycles between ECs with the miRNA:3′-adaptor ratio of 1:6 on one hand and ECs established with the miRNA:3′-adaptor ratio of 1:0.6 or 1:60 on the other hand. As in Table 1, we considered the top 800 ECs that were higher than 0.01*.

In summary, the expression of miRNAs in sequencing profiles does not reflect miRNA abundance in biological samples. Importantly, ECs appear slightly impacted by the number of amplification cycle and miRNA:3′-adaptor ratio. ECs can therefore be used as correcting factors for profiles established from biological samples displaying substantial differences in miRNA quantities.

### Correcting miRNA Expression Distortions

**Figure 4:**
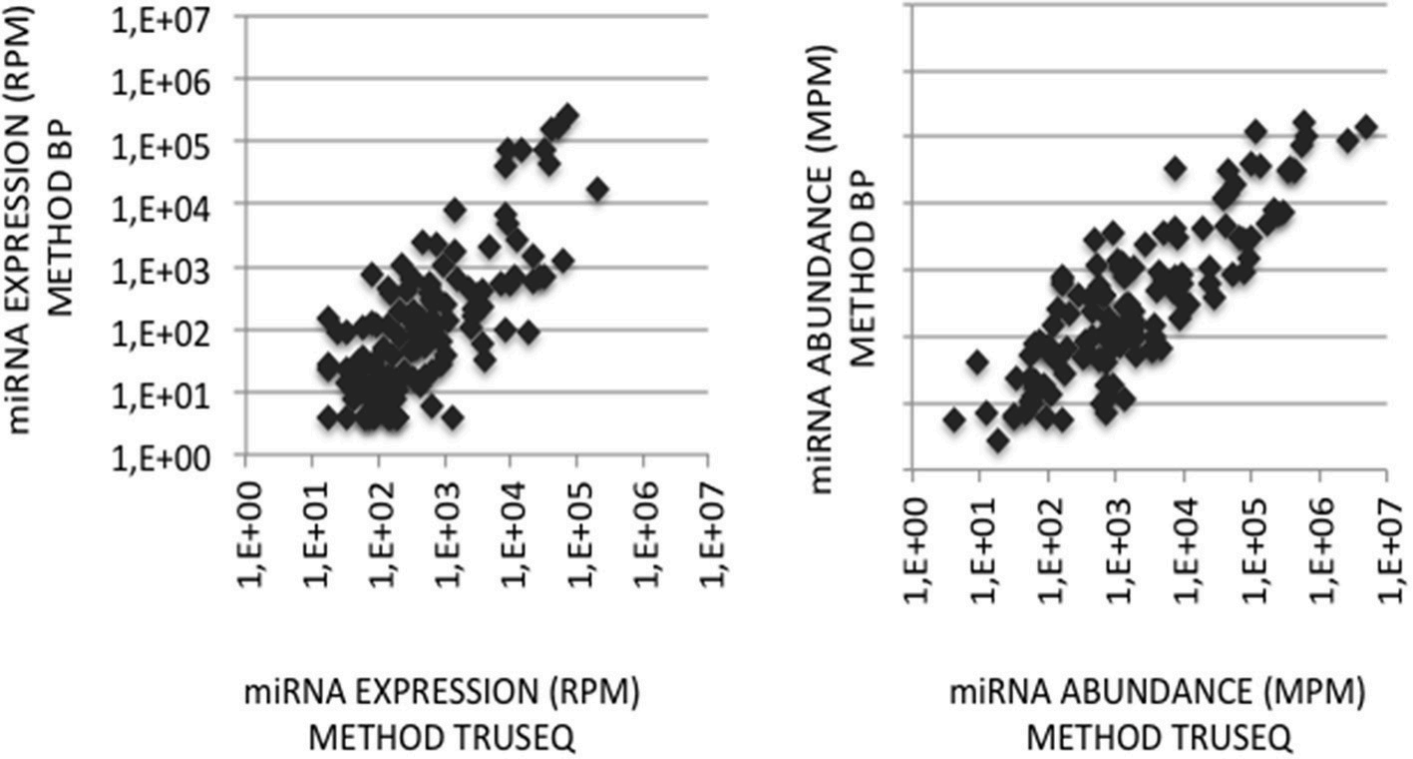
miRNA quantification in cerebellum. **(Left Graph)** miRNA expression profiles were established following the sequencing of two cDNA libraries constructed from the same cerebellum RNA sample and Illumina-based protocol but with 3′-adaptors RA3 (current TruSeq protocol) or BC8 (previous protocol). miRNA expressions in the BC8 profile were plotted against miRNA expressions in the RA3 profile. Data are expressed in Reads per Million (RPM). Both variables display a Pearson coefficient of correlation R^2^ of 0.54. **(Right Graph)** miRNA abundances in the cerebellum sample were calculated from each miRNA expression profile corrected with its corresponding ECs/correcting factors. miRNA abundances in the sample obtained using ECs/correcting factors BC8 were plotted against miRNA abundances calculated using ECs/correcting factors TruSeq. Data are expressed in Molecules per Million (MPM). Both variables display a Pearson coefficient of correlation R^2^ of 0.71. X- and Y-axes are drawn using a log_10_ scale.

**Figure 5:**
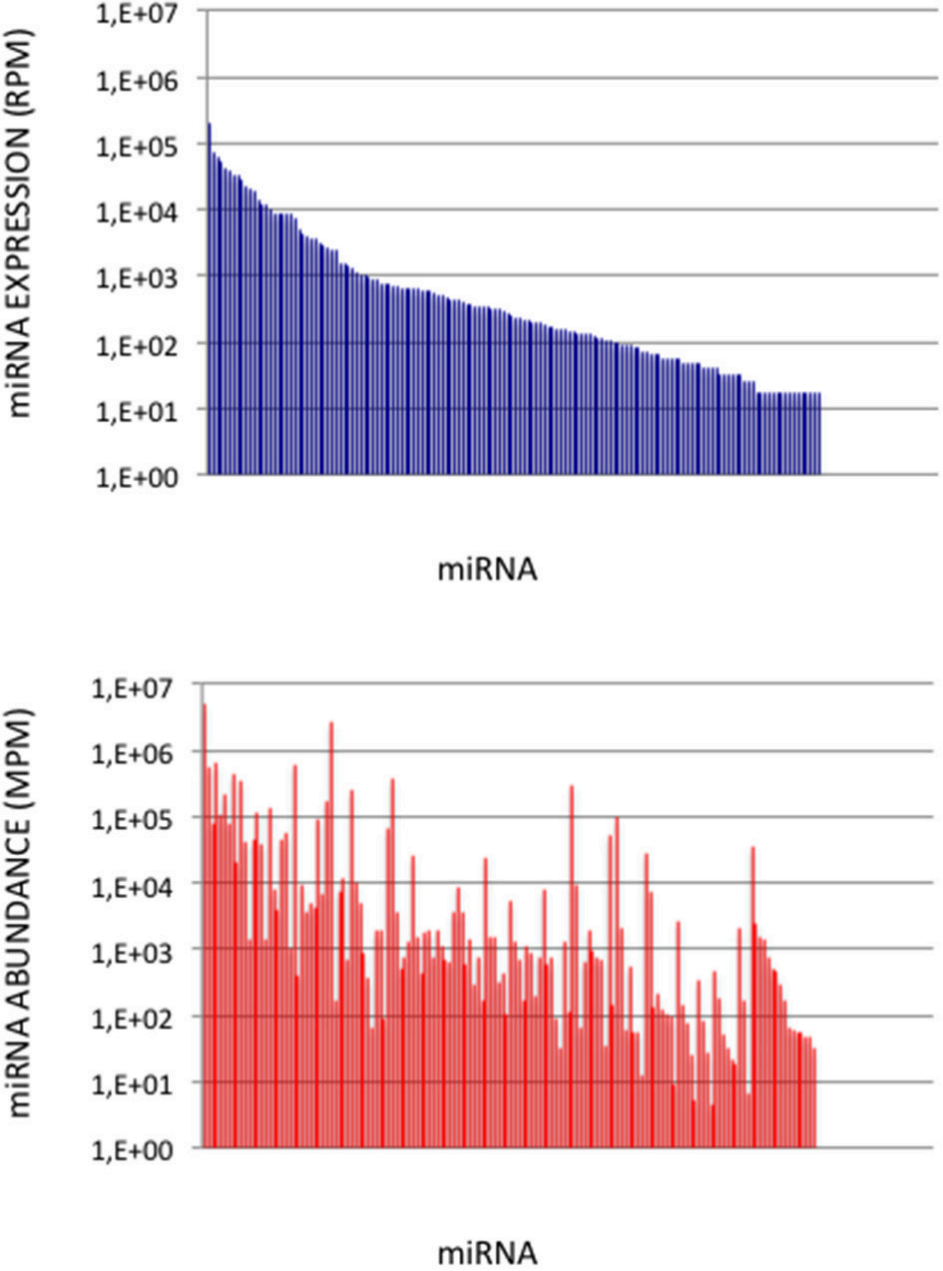
miRNA Abundances in Cerebellum. **(Upper Graph)** miRNA expressions ordered by decreasing values in the cerebellum expression profile. Data are expressed in Reads per Million (RPM). **(Lower Graph)** miRNA abundances in the cerebellum sample. miRNAs are ordered as in the upper graph. Data are expressed in Molecules per Million (MPM).

**Figure 6:**
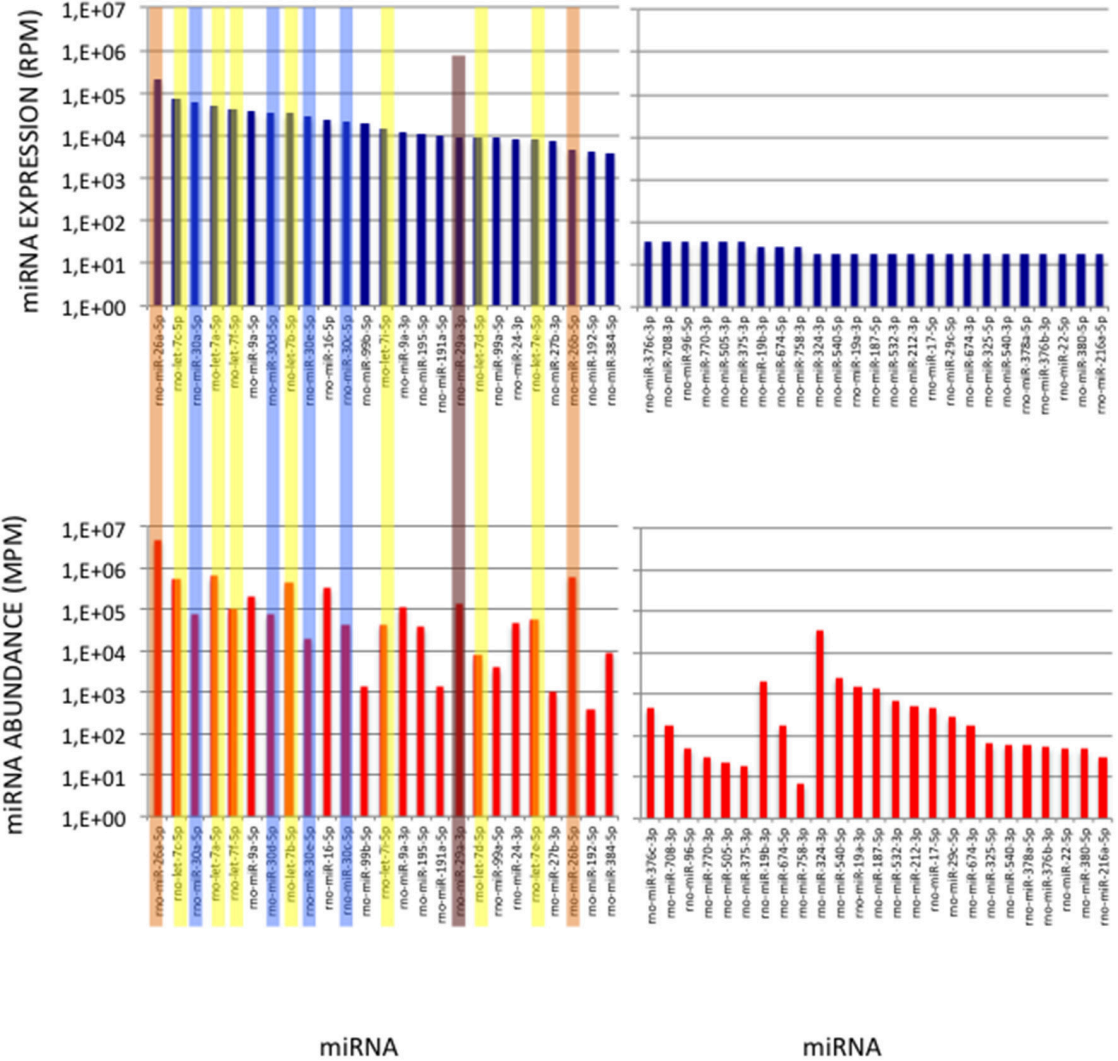
miRNAs of High Expression in the Cerebellum profile. **(Upper Graphs)** Expressions of the first and last 25 miRNAs in the cerebellum expression profile are shown, ordered by decreasing values. Data are expressed in Reads per Million (RPM). **(Lower Graphs)** Corresponding abundances in the cerebellum sample. miRNAs are ordered as in the upper graphs. Data are expressed in Molecule per Million (MPM). Five of the 25 more expressed miRNAs (>38,000 RPM) and 8 of the 25 less expressed miRNAs (< 100 RPM) in the expression profile turn to display similar abundances (400 < MPM < 4,000) in the sample. Members of the let-7, miR-26, miR-29, and miR-30 families are pictured in yellow, orange, purple, and blue, respectively.

**Figure 7:**
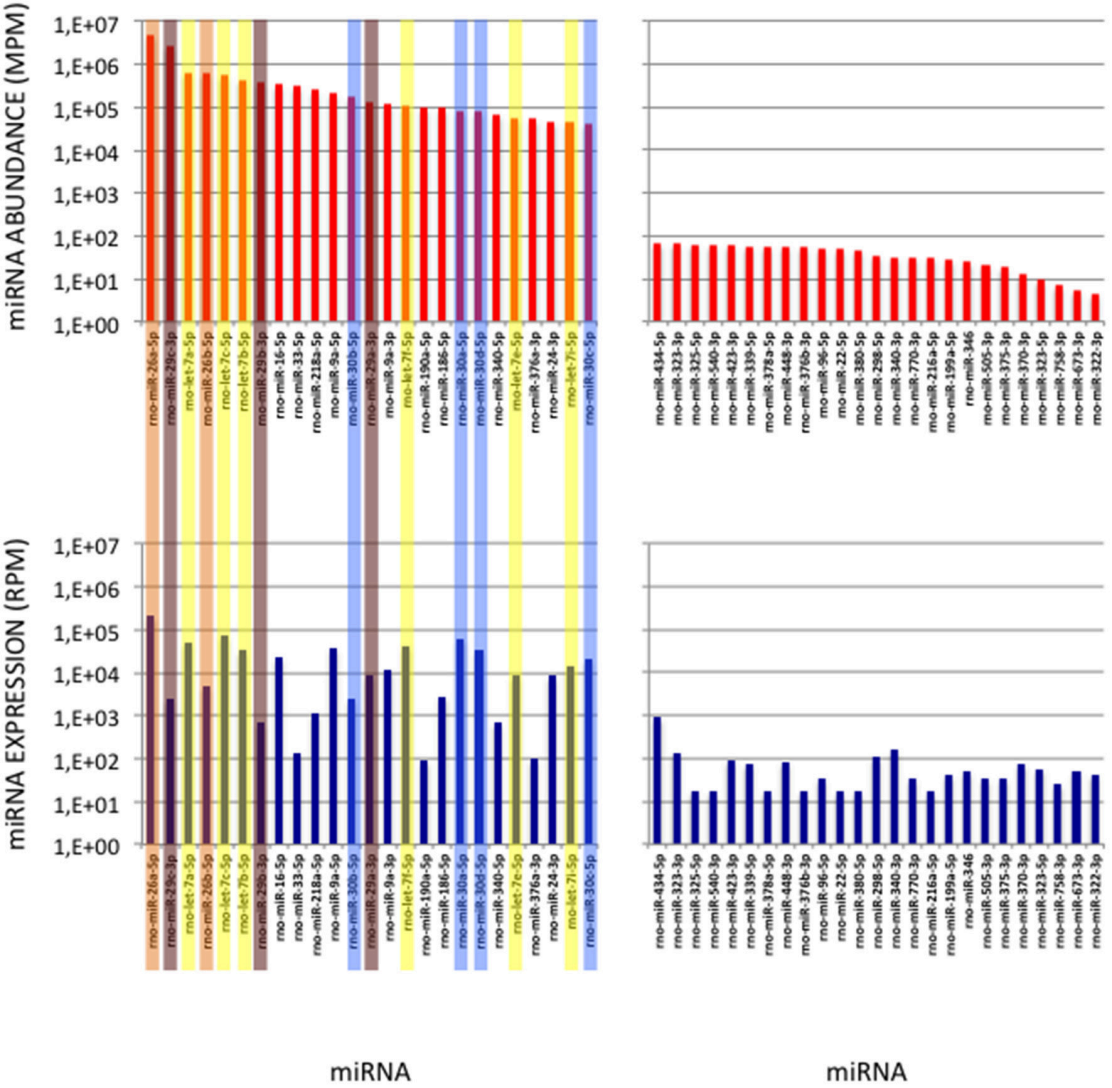
miRNAs of High Abundance in the Cerebellum Sample. **(Upper Graphs)** Abundances of the first and last 25 miRNAs in the cerebellum sample are shown, ordered by decreasing values. All 5p-members of the miR-26 and miR-29 families appear highly abundant (ranks < rank 14). Data are expressed in Molecules per Million (MPM). **(Lower Graphs)** Corresponding expressions in the cerebellum expression profile. Data are expressed in Reads per Million (RPM). miRNAs are ordered as in the upper graphs. Members of the let-7, miR-26, miR-29 and miR-30 families are pictured in yellow, orange, purple and blue, respectively.

To validate the use of correcting factors for reducing expression biases, we took advantage of a previously reported experiment illustrating the strong adaptor dependency of miRNA expression profiles (Baroin-Tourancheau et al., 2016). In this experiment, we had shown that two cDNA libraries constructed from the same rat adult cerebellum RNA sample and Illumina protocol, but with two different 3′-adaptors, provided two miRNA expression profiles highly different. One of them, RA3, is the 3′-adaptor currently used in TruSeq Illumina protocols. The other one, BC8, that was used in previous Illumina protocols, displays an unrelated sequence. We determined the ECs/correcting factors characterizing the use of BC8 adaptor in the same way that above (Supplemental Table 4). We then converted miRNA expressions of the RA3- and BC8-cerebellum profiles into miRNA abundances, each with their corresponding ECs/correcting factors (Figure 4). A much closer relationship was observed between both miRNA expression profiles after correction than before correction. The Pearson correlation coefficient R^2^ increased from 0.54 before correction to 0.71 after correction demonstrating that the use of the ECs/correcting factors greatly improved the resemblance of the expression profiles to the miRNA cerebellum content. We used those data to identify the abundance of more than 200 miRNAs (Figures 5–7). miRNAs of the let-7, miR-26, miR-29, miR-30 families appeared as the most abundant miRNAs of the rat adult cerebellum. miR-26a-5p, in particular, ranked as the first top expressed miRNA (see Figure 7). As miR-26b-5p also was one of the more abundant miRNAs (rank 4), the whole miR-26 family appeared as an important marker of the rat adult cerebellum. Three miRNAs of the miR-29 family, i.e., miR-29a-3p, −29b-3p, and −29c-3p ranked as the 2nd, 7th, and 13th more abundant miRNAs thereby defining the miR-29 family as a second marker of the cerebellum of adult male rat. Of note, in non-corrected Illumina-based profiles, miR-29a-3p, −29b-3p, and −29c-3p only ranked as the 17th, 32th, and 47th more expressed miRNAs.

## Discussion

The relatively limited number of the miR genes (miRNA-specifying genes) allied to the huge sequencing capacity of the Illumina technology lead miRNA-Seq to become more and more prevalent in fundamental research as well as in clinical studies using miRNAs as biomarkers. The latest version (release 22) of the miRBase database (Kozomara and Griffiths-Jones, 2014), a central repository for miRNAs, lists sets of 258, 1,234, and 1,917 miRNAs in the fly, mouse, and human genomes, respectively. The Illumina sequencing technology that currently generates hundreds millions of reads of 40–100 nucleotides per flow cell lane is particularly well adapted to explore miRNA transcriptomes. Thirty to Forty samples can be sequenced at a sequencing depth of 2–3 millions reads in a single lane allowing to increase sample size.

If there is no question that miRNA-Seq is very powerful to measure expression changes in miRNA populations, this technology suffers inherent RNA ligation biases that greatly affect the representativeness of each miRNA in cDNA libraries (Fuchs et al., 2015). Here we describe a method that uses a current TruSeq/Ilumina protocol and the equimolar mixture of synthetic miRNAs of the miRXplore Universal Reference to quantify ligation biases and provide Tables of ECs/correcting factors that allow miRNA abundances in biological samples to be calculated. The miRXplore Universal Reference annotated 400, 503, and 569 miRNAs of the rat, mouse and human miRBase database, respectively. Note that our data are valuable for many genomes as many miRNA sequences have been conserved during evolution. For miRNAs absent from the miRXplore Universal Reference, our method can be extended to new miRNAs by complementing the miRXplore Universal Reference with RNA oligonucleodides ordered on purpose. The use of ECs/correcting factors deeply improves the valid selection of miRNAs of interest and design of experiments in physiological/physiopathological research.

When calculating ECs, we observed a five-order magnitude difference between the most and the least frequently detected miRNAs. Fuchs and co-authors identified RNA secondary structures of the ligated substrates as the major contributor (2015). Very interestingly, they could identify distinct secondary structural features favoring or disfavoring adaptor ligations. miRNAs with paired-bases at their 5′ and/or 3′ ends were less accessible to the ligation reaction and thus under-represented in the cDNA libraries. Our sequencing results provide expression data that should be analyzed in light of their secondary structural properties in order to comfort and enrich these predictive rules. Indeed, when comparing the content of cDNA libraries generated from the miRXplore Universal Reference with the RA3 or BC8 3′-adaptors, there was a common set of RNAs poorly or not captured by any of them (EC < 1E-3). In order to limit ligation-based artifacts, strategies have been proposed that rely on the design of versatile adaptors carrying randomized sequences (Sorefan et al., 2012; Fuchs et al., 2015). It is unclear how efficiently such adaptors will capture those RNA sequences.

More than 90% of the reads mapped to the full-length miRNA reference sequences. About 10% of the reads mapped to sequences shorter by one or a few nucleotides, identifying molecules that probably result from interrupted oligonucleotide chemical syntheses. While the reference sequences identify canonical miRNAs, shorter sequences identify short isomiRs. Differences in terminal nucleotides did not seem to promote large differences in adaptor-ligation efficiencies: truncated miRNAs are expected to be minor products in the miRXplore Universal Reference and shorter sequences appeared as minor products in expression profiles. Further experiments using the miRXplore Universal Reference complemented with various amounts of isomiRs will allow precise quantification of their ECs/correcting factors.

Our goal in this work was to develop a simple method to quantify biases in miRNA expression profiles generated from cDNA libraries constructed with currently used TruSeq adaptors and characterize miRNA relative abundance in biological samples. We showed that, at a given miRNA:3′ adaptor ratio, the number of amplification cycles differently impacted ECs of rarely and frequently detected miRNAs. CVs of ECs slightly increased from 0.1 up to 0.6. An important point was the relative robustness of ECs against the miRNA:3′-adaptor ratio, the most fluctuating parameter in standardized protocols. By using ratios of miRNA:3′-adaptor ranging over two orders of magnitude, we showed that CVs of ECs were steady with a mean value of 0.3 and a standard deviation value of 0.05. Even large differences in the amount of miRNAs used for cDNA library construction, a parameter difficult to control when working with minute biological samples, has a moderate impact. As a best-practice when working with biological samples, we recommend to purify RNAs of 16–40 nucleotides from 1 to 10 ug of total RNA, use purified RNAs of 16–40 nucleotides corresponding to 0.3 to 1 ug of total RNA, a constant number of PCR amplification cycles lower than 20 and the protocol described in the Materials and Methods section (see Supplemental Figure 1).

We used our data to characterize miRNA abundance in the cerebellum of a male adult rat. We identified miRNAs of the let-7, miR-26, miR-29, miR-30 families as the more abundant miRNAs and markers of this brain area. The miR-29 family is expressed across various tissues and enriched in mouse brain, more particularly in the cerebellum and hippocampus (Bitetti et al., 2018). The miR-29 family received much attention those last years especially in the cerebellum and the defective expression of miR-29b was shown to impair proper neuron maturation. Our data show that the functional importance of the miR-29 family in the cerebellum is associated with a predominance of mature miR-29 miRNAs in the miRNA cerebellum population. Our data also revealed that the miR-26 family is expressed at a level similar to that of the miR-29 family. To our knowledge, the function of the miR-26 family in the cerebellum has not received the attention that its abundance deserves.

## Conclusion

In many expression profiles arisen from miRNA-Seq studies, there is a trend to abusively consider miRNA expression ranks as biologically meaningful. It is important to stress that miRNA-enriched cDNA libraries have been constructed with adaptors that are prone to ligation biases and that this only allows miRNA expression differences between samples to be identified. New adaptors that reduce ligation biases, thus leading to miRNA-derived cDNA libraries closer to biological samples, have been designed. However, as large biases are still produced, calculation of ECs/correcting factors in current TruSeq/Illumina protocols appear as a strategy of choice for evaluating miRNA abundance in biological samples, valid selection of miRNAs of interest and sound design of experiments in physiological and physiopathological researches.

## Author Contributions

AB-T and LA conceived and designed the experiments. AB-T, YJ, and LA performed the experiments. XB developed the algorithms. AB-T and LA analyzed the data. AB-T and LA wrote the paper. All authors reviewed the manuscript.

### Conflict of Interest Statement

The authors declare that the research was conducted in the absence of any commercial or financial relationships that could be construed as a potential conflict of interest.
