# Supplementary figures and images for: Evaluating and Correcting Inherent Bias of microRNA Expression in Illumina Sequencing Analysis
Source: Front Mol Biosci. 2019 Apr 24;6:17. doi: 10.3389/fmolb.2019.00017 (PMC6491513; doi:10.3389/fmolb.2019.00017)

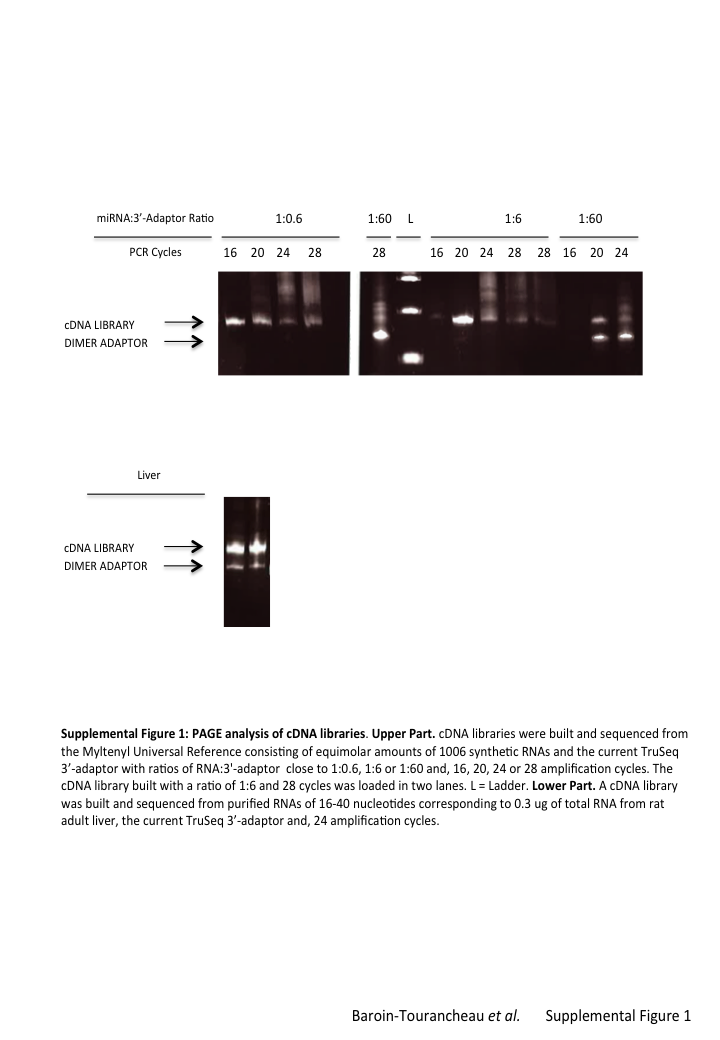

Supplement: Supplementary file 5 [file Image_1.TIFF]

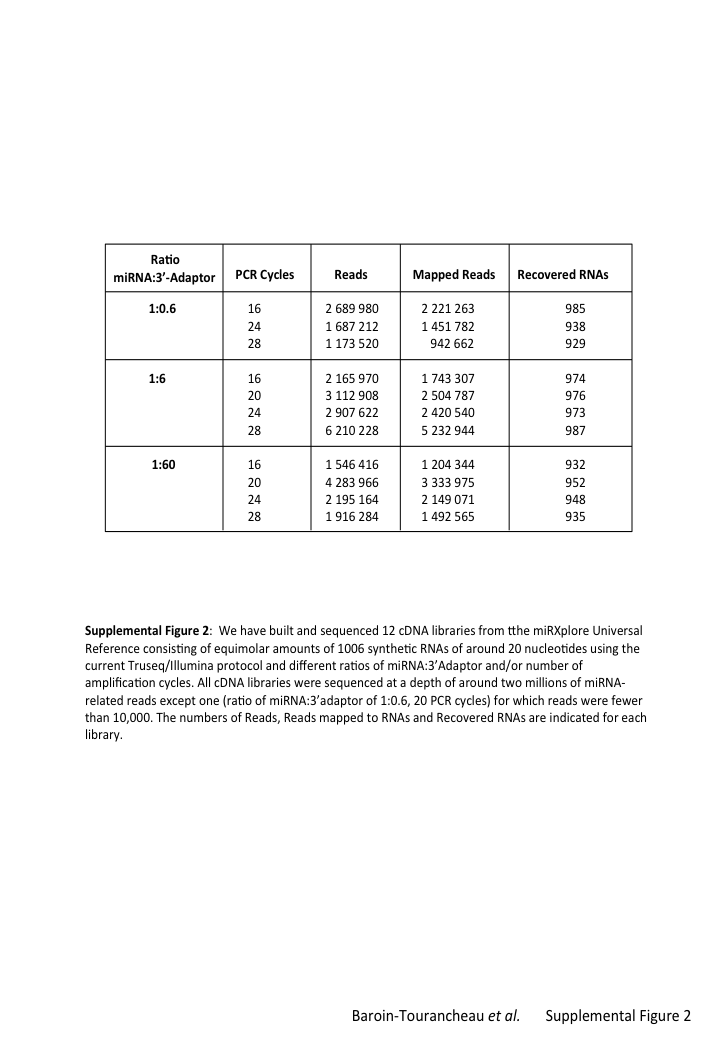

Supplement: Supplementary file 6 [file Image_2.TIFF]

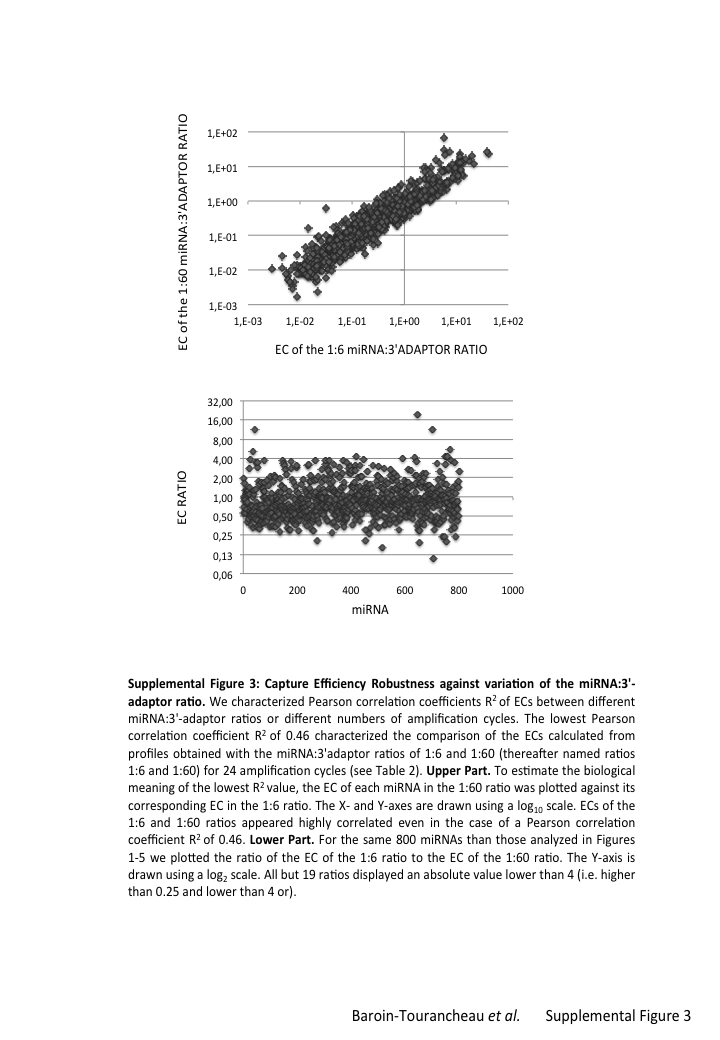

Supplement: Supplementary file 7 [file Image_3.TIFF]
